# Karyological and molecular analysis of three endemic loaches (Actinopterygii: Cobitoidea) from Kor River basin, Iran

**Published:** 2015-01

**Authors:** Hamid Reza Esmaeili, Zeinab Pirvar, Mehragan Ebrahimi, Matthias F. Geiger

**Affiliations:** 1 Department of Biology, College of Sciences, Shiraz University, Shiraz, Iran; 2 Zoological Research Museum Alexander Koenig, Leibniz Institute for Animal Biodiversity, Adenauerallee, Germany

**Keywords:** Loaches, Phylogenetic relationships, COI barcode region, Idiogram, Iran

## Abstract

This study provides new data on chromosomal characteristics and DNA barcoding of three endemic loaches of Iran: spiny southern loach *Cobitis linea* (Heckel, 1847), Persian stream loach *Oxynoemacheilus persa* (Heckel, 1848) and Tongiorgi stream loach *Oxynoemacheilus tongiorgii* (Nalbant & Bianco, 1998). The chromosomes of these fishes were investigated by examining metaphase chromosome spreads obtained from epithelial gill and kidney cells. The diploid chromosome numbers of all three species were 2n=50. The karyotypes of *C. linea* consisted of 4M + 40SM + 6ST, NF=94; of *O. persa* by 20M + 22SM + 8ST, NF=90 and of *O. tongiorgii* by 18M + 24SM + 8ST, NF= 92. Sex chromosomes were cytologically indistinguishable in these loaches. Maximum likelihood-based estimation of the phylogenetic relationships based on the COI barcode region clearly separates the three Iranian loach species of the Kor River basin. All species distinguished by morphological characters were recovered as monophyletic clades by the COI barcodes. The obtained results could be used for population studies, management and conservation programs.

## INTRODUCTION

The confirmed freshwater ichthyofauna of Iran are represented by 202 species in 104 genera, 28 families, 17 orders and 3 classes found in 19 different basins [1]. The most diverse order is the Cypriniformes with 120 confirmed species (59.4%) including Cyprinidae with 93 confirmed species (46.0%), Nemacheilidae with 22 species (10.9%) and Cobitidae with 5 species (2.5%) [1]. However, a few new and exotic fishes have been recently reported from inland waters of Iran, increasing the number of confirmed species to more than 220 [2-8]. From a cytogenetic point of view, few of these freshwater fish species have been chromosomally characterized [9-12] and cobitid (Cobitidae) and nemacheilid (Nemacheilidae) loaches have not been completely accounted for so far [13]. 

The Cobitidae family, sometimes called sting-loaches (spiny loaches), is found in Eurasia and Morocco and has about 26 genera with about 177 species [14]. Four Cobitidae species have been recorded from Iran [1, 13]. Loaches of the Nemacheilidae family are a characteristic element of the Eurasian ichthyofauna and occur in nearly every running water. About 30 genera and 720 nominal species are presently known, most of them from South and Southeast Asia. However, a great number of taxa remain to be described [15].

The same situation is observed in Iran and new species are being described whose taxonomic statuses are being reviewed [5, 16-19] mostly based on their morphology and anatomy (gas bladder capsule, gut). The application of non-morphological methods such as cytogenetic and molecular studies may provide a complementary data source for more accurate and precise identification of these fishes. These types of studies have received considerable attention in recent years [19, 20-23]. Fish chromosome data have great importance in studies concerning evolutionary systematics, aquaculture, mutagenesis, genetic control and the rapid production of inbred lines [22, 24]. The study of karyotype is also important in aquaculture in connection with the use of chromosome manipulation techniques, including the induction of polyploidy, gynogenesis, androgenesis and inter or intra-species hybridization [25, 26]. About 3425 freshwater and marine fish species have been reviewed in this respect [22] which is about 10.5% of the 32,700 described fish species. Moreover, recent molecular systematics has enabled the re-assessment of many fish taxa and provided phylogenetic hypotheses for them. In this context, DNA barcoding using short, standardized DNA sequences to identify species by using primers that are applicable to the broadest possible taxonomic group have generated novel insights from faunal assessments. Our main goals are to contribute to the understanding and exploring of cytogenetical data (i.e., diploid chromosome numbers, description of karyotypes, idiograms) and phylogenetic relationships of three endemic loaches of the Kor River basin based on the COI barcode region in order to help future taxonomical and genetic studies.

## MATERIALS AND METHODS

**Figure 1:**
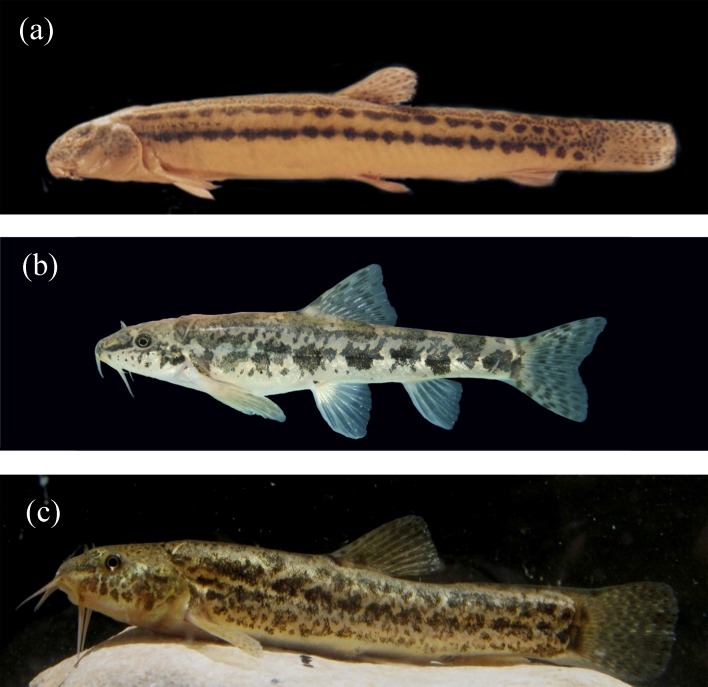
Endemic loaches of the Kor River basin in Iran. a, *Cobitis linea*; b, *Oxynoemacheilus* *persa*; c, *Oxynoemacheilus tongiorgii*.

*Cobitis* *linea*, *Oxynoemacheilus persa* and *Oxynoemacheilus tongiorgii *specimens (Fig. 1) ?′?′^27^] was followed. Colchicine solution was prepared with 0.005 g in a 20 ml physiological serum. The fish were injected intraperitoneally with 0.02 ml of colchicine per gram of body weight using an insulin syringe and taken back to the aquarium for 4-5 hours. They were then anaesthetized using MS222, and their gill filaments and kidneys were removed and placed in hypotonic 0.36% KCl solution for 45 min in room temperature (25˚C). After adding 2-3 drops of fresh and cold Carnoy fixative (1: 3, Acetic acid: Methanol), the solutions were centrifuged for 10 min at 1000 rpm. The supernatants were then discarded and 5ml fresh and cold fixative was added to the sediments, mixed thoroughly and left for 1 hour. The fixation and centrifugation stages were repeated twice. The suspensions now were trickled to cold slides from a height of almost 2 meters. These slides were stained with 10% Giemsa for 20 min. Chromosomes were observed, selected and photographed by an Olympus light microscope mounted with a camera. Karyotypes were prepared by arranging chromosomes in pairs by size. For each chromosome, the average lengths of short and long arms, the arm ratio (the ratio of the length of the long arm to the short arm or r value) and the centromeric index (CI, expressed as the ratio of the length of the short arm to total chromosome length) were calculated and chromosomes were classified according to Levan et al.’s (1964) criteria [28]. Fundamental number (NF) was expressed as twice the atelocentric number plus the number of telocentric chromosomes. 

**DNA extraction and PCR: **Genomic DNA was extracted using Macherey & Nagel NucleoSpin® Tissue kits following the manufacturer’s protocol on an Eppendorf EpMotion® pipetting-roboter with vacuum manifold. The standard vertebrate DNA barcode region of the COI (cytochrome c oxidase subunit 1) was amplified using an M13 tailed primer cocktail including FishF2_t1 (5’TGT AAA ACG ACG GCC AGT CGA CTA ATC ATA AAG ATA TCG GCA C3’), FishR2_t1 (5’CAG GAA ACA GCT ATG ACA CTT CAG GGT GAC CGA AGA ATC AGA A3’), VF2_t1 (5’TGT AAA ACG ACG GCC AGT CAA CCA ACC ACA AAG ACA TTG GCA C3’) and FR1d_t1 (5’CAG GAA ACA GCT ATG ACA CCT CAG GGT GTC CGA ARA AYC ARA A3’) [29]. Sequencing of the ExoSAP-IT (USB) purified PCR product in both directions was conducted at Macrogen Europe Laboratories with forward sequencing primer M13F (5’GTA AAA CGA CGG CCA GT3’) and reverse sequencing primer M13R-pUC (5’CAG GAA ACA GCT ATG AC3’). 

**Molecular data analysis: **Data processing and sequence assembly was carried out in Geneious [30] and Muscle algorithm [31] was chosen to create a DNA sequence alignment. Modeltest [32], implemented in the MEGA 6 software [33], was used to determine the most appropriate sequence evolution model for the given data, treating gaps and missing data with the partial deletion option under 95% site coverage cutoff. The model with the lowest BIC (Bayesian Information Criterion) scores was considered to best describe the substitution pattern. According to Modeltest, the Tamura-Nei model [34] with discrete Gamma distribution (5 categories (+G, parameter = 0.4292)) best represented the COI alignment, and was used to estimate the evolutionary history. We generated maximum likelihood phylogenetic trees with 500 bootstrap replicates to explore species phylogenetic affinities. As an appropriate outgroup to root the constructed phylogenetic hypothesis, we included the loach *Misgurnus*. 

**Materials used for molecular COI analysis: **Twelve loach specimens from the Kor River basin of Iran: *Oxynoemacheilus persa*: Kor_ Iran_1983_Ex91E1; KP050538; *Oxynoemacheilus persa*: Kor_Iran_1983_Ex91E2; KP050531; *Oxynoemacheilus persa*: Kor_Iran_1983_Ex91E3; KP050533; *Oxynoemacheilus persa*: Kor Iran_1983_Ex91E4; KP050529; *Oxynoemacheilus tongiorgii*:* Kor_Iran_6_Ex82E10; KP050537*; *Oxynoemacheilus tongiorgii*: Kor_Iran_6_Ex82E11; KP050532; *Oxynoemacheilus tongiorgii*: Kor_Iran_6_Ex87A2; KP050536; *Oxynoemacheilus tongiorgii*: Kor_ Iran_6_Ex87A3; KP050535; *Oxynoemacheilus tongiorgii*: Kor_Iran_6_Ex87A4; KP050534; *Oxynoemacheilus tongiorgii*: Kor_Iran_6_Ex87A5;KP050540 *Cobitis linea*: Kor_Iran_1982_Ex82A7; KP050530; *Cobitis linea*: Kor_Iran_1982_Ex82A6; KP050539.

**Comparative material from GenBank:** *Oxynoemacheilus panthera*_KJ554017; *Oxynoemacheilus namiri*_KJ553891; *Oxynoemacheilus angorae*_KJ553966; *Oxynoemacheilus* *angorae*_KJ553824; *Oxynoemacheilus anatolicus*_KJ443916; *Seminemacheilus ispartensis*_KJ554948; *Seminemacheilu*s sp._KJ554960; *Cobitis elongatoides*_HQ961002; *Cobitis vardarensis*_HQ600718; *Cobitis taenia*_KJ128459;* Cobitis taenia*_KJ128460; *Cobitis lutheri*_HQ536324; *Misgurnus fossilis*_JQ011436; *Misgurnus fossilis*_JQ011436.

## RESULTS AND DISCUSSION

**Figure 2:**
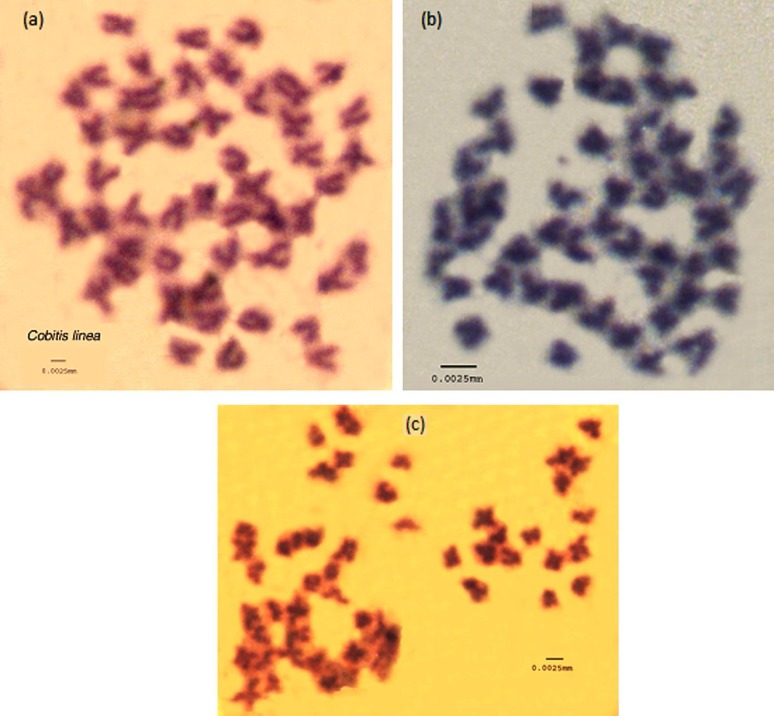
Giemsa stained chromosome spreads of three loaches from Iran. . a, *Cobitis linea*; b, *O. persa *; c, *O. tongiorgii*

**Figure 3:**
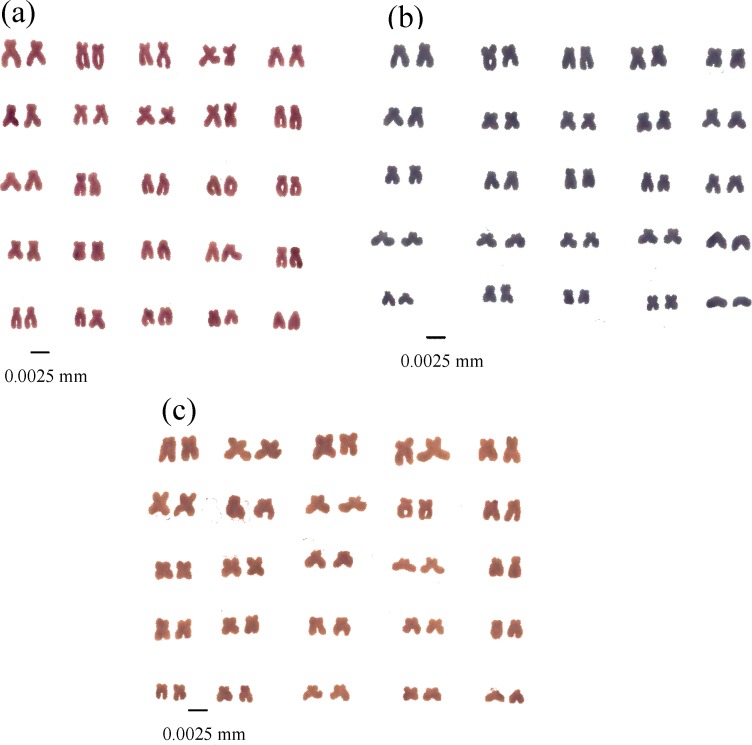
Karyotypes of three loaches from Iran. a, *C. linea*; b, *O. persa *; c, *O. tongiorgii*

**Figure 4:**
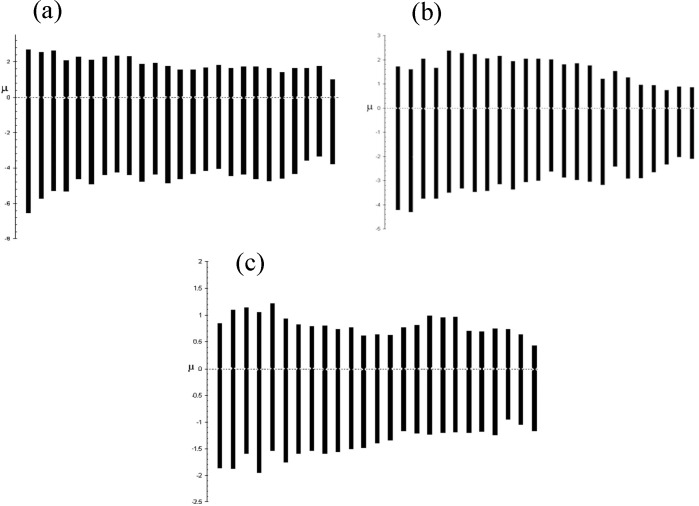
Haploid idiograms of three species of loaches from Iran. a, *C. linea*; b, O.* persa *; c, *O. tongiorgii*.

Metaphase spreads of the three species are given in Fig. 2. Diploid chromosome numbers of all three species were 2n=50 (Fig. 3). Quantitative data of the different measurements used to classify chromosomes and idiograms are given in table I and figure 4. The karyotypes consisted of 2 pairs of metacentric, 20 pairs of submetacentric and 3 pairs of subtelocentric chromosomes (4m + 40sm + 6st) in *C. linea*; 10 metacentric, 11 submetacentric and 4 subtelocentric (20m +22sm + 8st) in *O. persa* and 9 metacentric, 12 submetacentric and 4 subtelocentric (18m +24sm + 8st) in *O. tongiorgii*. The arm numbers were 94, 90 and 92 in *C. linea*, *O. persa* and *O. tongiorgii* respectively. Sex chromosomes were cytologically indistinguishable in these loaches.

**Figure 5:**
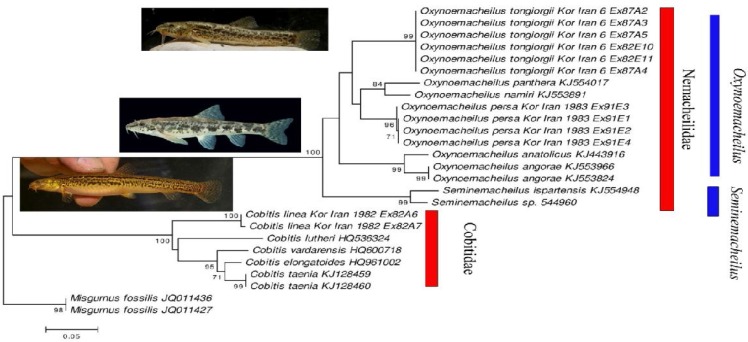
Maximum Likelihood (ML) estimation of the phylogenetic relationships based on the mitochondrial COI barcode region. Nucleotide positions with less than 95 % site coverage were eliminated before analysis. Numbers of major nodes indicate bootstrap values above 65% from 500 pseudo-replicates. All branch lengths are drawn to scale and give number of substitutions per site. The photos show *O. tongiorgii*, O.* persa *, and *C. linea*

Maximum likelihood based estimation of the phylogenetic relationships based on the COI barcode region clearly separated the three Iranian loach species from the Kor drainage (Fig. 5). All species distinguished by morphological characters are recovered as monophyletic clades by the COI barcodes. K2P distances between (sympatric) species in Kor drainage loaches were found for *Cobitis linea* to *Oxynoemacheilus tongiorgii* (41.3 % K2P), *Cobitis linea* to *Oxynoemacheilus persa *(38.4% K2P) and *Oxynoemacheilus persa *to *Oxynoemacheilus tongiorgii* (10.3 % K2P). Smallest K2P distances between genera were found for *Oxynoemacheilus* to *Seminemacheilus* (8.1 % K2P) and *Oxynoemacheilus* to *Cobitis* (38.4 % K2P).

The resolution of the mitochondrial COI gene fragment does not, however, allow for unequivocal inference of sister species relationships and the restricted outgroup sampling available for this study sets clear constraints on the phylogenetic interpretation of the results. 

In many vertebrate groups, the study of karyotypes and genome size has contributed, along with analyses of mitochondrial and nuclear gene sequences, to the solution of various challenges in biology, systematics and evolution [22]. However, in fishes, which are the most diverse of all vertebrate groups, higher taxa have been traditionally classified largely by morphology and paleontology, with a much smaller input of cytogenetic information., This is partly due to the fact that karyotypes can be obtained only from living specimens, tissues, or cells, which makes it challenging to study the karyotypes of fishes that are difficult to collect alive (e.g. deep-sea fishes). Nevertheless, even fresh material provides no guarantee that reliable chromosome figures can be obtained easily [22]. Karyotypes are descriptions of the number and morphology of chromosomes. The number of chromosomes per cell seems to be a rather conservative characteristic and may thus be used as an indicator of the closeness of species’ interrelationships within families [35]. The diploid chromosome number of fishes varies from 2n= 22-26 in some species of an Antarctic fish group [36], to 2n=240-260 in some anadromous Acipenseridae which show several microchromosomes [37].

**Table 1:** Long arm length, LA (µm); short arm length, SA(µm); total arm length, TA (µm); arm ratio, AR; centromeric index, CI and chromosome type, CT of three endemic loaches of Iran

**C.linea**					**O.persa**						**O.tangiorgii**							
No.	LA	SA	TA	AR	CI	CT	LA	SA	TA	AR	CI	CT	CT	LA	SA	TA	AR	CI
1	6.53	2.69	9.21	2.43	29	Sm	1.85	.59	2.34	3.13	.25	St	St	1.97	.63	2.60	3.12	.24
2	5.72	2.51	8.22	2.28	.30	Sm	1.78	.98	2.76	1.82	.36	Sm	St	1.92	1.09	3.01	1.76	.36
3	5.32	2.64	7.95	2.02	.33	Sm	1.55	.70	2.25	2.21	.31	Sm	St	1.59	1.13	2.72	1.41	.42
4	5.48	2.11	7.58	2.6	.28	Sm	1.52	.49	2.01	3.10	.24	St	M	1.97	1.05	3.02	1.88	.35
5	4.92	2.1	7.01	2.35	.30	Sm	1.35	.91	2.26	1.48	.40	M	Sm	1.44	1.34	2.78	1.07	.48
6	4.64	2.29	6.94	2.03	.33	Sm	1.12	.90	2.02	1.24	.45	M	M	1.78	.93	2.71	1.91	.34
7	4.97	1.76	6.73	2.83	.26	Sm	1.27	.79	2.06	1.61	.38	M	Sm	1.59	.82	2.41	1.94	.34
8	4.43	2.28	6.71	1.94	.34	Sm	1.39	.69	2.08	2.01	.33	Sm	Sm	1.54	.78	2.32	1.97	.34
9	4.86	1.58	6.7	1.63	.28	M	1.34	.66	2.00	2.03	.33	Sm	M	1.59	.80	2.39	1.99	.33
10	4.29	2.4	6.69	1.78	.36	Sm	1.29	.62	1.91	2.08	.32	Sm	Sm	1.51	.82	2.33	1.84	.35
11	4.36	2.3	6.69	1.91	.34	Sm	1.39	.62	2.01	2.24	.31	Sm	Sm	1.51	.82	2.33	1.84	.35
12	4.74	1.62	6.36	2.93	.25	Sm	1.33	.59	1.92	2.25	.31	Sm	Sm	1.99	.60	2.59	3.31	.23
13	4.57	1.74	6.31	2.63	.28	Sm	1.59	.50	2.09	3.11	.24	St	St	1.42	.63	2.05	2.25	.31
14	4.36	1.94	6.3	2.25	.31	Sm	1.23	.69	1.92	1.78	.36	Sm	Sm	1.16	.62	2.04	2.29	.30
15	4.57	1.61	6.18	2.85	.26	Sm	1.19	.69	1.88	1.72	.37	Sm	Sm	1.22	.76	1.92	1.53	.40
16	4.44	1.7	6.14	2.61	.28	Sm	1.35	.62	1.97	2.18	.31	Sm	St	1.24	.78	2.00	1.56	.39
17	4.62	1.46	6.08	3.16	.24	St	1.21	.77	1.98	1.57	.39	M	St	1.18	1.09	2.33	1.14	.47
18	4.32	1.65	5.97	2.62	.28	Sm	1.13	.67	1.80	1.69	.37	Sm	Sm	1.21	.95	2.13	1.24	.45
19	4.59	1.35	5.94	3.41	.23	St	1.18	.74	1.92	1.59	.39	M	Sm	1.20	1.00	2.21	1.21	.45
20	4.05	1.85	5.9	2.2	.31	Sm	1.07	.79	1.86	1.35	.42	M	M	1.18	.70	1.90	1.71	.37
21	4.33	1.54	5.86	2.82	.26	Sm	1.04	.63	1.67	1.65	.38	M	Sm	1.35	.64	1.82	1.84	.35
22	4.16	1.68	5.83	2.48	.29	Sm	1.04	.68	1.72	1.53	.40	M	M	.94	.74	2.09	1.82	.35
23	3.69	1.7	5.36	2.15	.32	Sm	.95	.62	1.57	1.53	.39	M	St	1.05	.79	1.73	1.19	.49
24	3.34	1.98	5.32	1.69	.37	M	1.06	.56	1.62	1.89	.35	Sm	Sm	1.05	.66	1.71	1.59	.39
25	4.07	99	5.06	4.11	.20	St	2.13	.50	2.63	4.26	.19	St	Sm	1.58	.42	2.00	3.76	.21

According to our observations, the diploid chromosome numbers of all the endemic loach species were 2n= 50, being in conformation with the chromosome number of other species and genera of loaches. Klinkhardt et al., (1995) [38] and Arkhipchuk (1999) [39] reported the chromosome number of *Cobitis calderoni* (Bacescu, 1962), *C. granoei* Rendahl, 1935, *C. lutheri* Rendahl, 1935, *C. macroccana* Pellegrin, 1929, *C. taenia* Linnaeus, 1758 and also Niwaella delicata (Niwa, 1937) to be 2n= 50. Other balitorid species such as *Barbatula barbatula *(Linnaeus, 1758), *Acanthocobitis boti*a (Hamilton, 1822), *Triplophysa dorsalis *(Kessler, 1872), and *T. stoliczkai *(Steindachner, 1866) which have been cytologically investigated so far, have the diploid chromosome number 2n=50 [38, 39]. It can be concluded that the chromosome number in this group is conservative. Yet, in few species of Cobitoidea, the diploid chromosome number is reported to vary from 2n=48 to 2n=94 [38, 39]. The chromosome number of *C. biwae *(Jordan & Snyder, 1901) and* Cobitis takatsuensis *(Mizuno, 1970) was reported to be 2n= 48 [39], for *C. matsubarai* (Okada & Ikeda, 1939) it was 2n= 86, 94 [40], and for *C. taenia* (Linnaeus, 1758) it was 2n=50, 75, 86, 94 [39, 38]. It could be suggested that the most common diploid chromosome number is 2n=50, which is the modal number in loach fishes. However, a cytological indication of polyploidy has been noted in some loach species. Polyploidy has been also noted among members of the Salmonidae, Cyprinidae, Cobitidae and Catostomidae families [22, 41].

When interpreting karyotypic evolution, it is often assumed that the primitive fish karyotype consists of 48 rods from which the karyotypes of all existing fish forms have been derived [41], but this issue is yet to be resolved. The discovery of 48 rather large acrocentric chromosomes in the Pacific hag fish, *Eptatretus stouti*i, belonging to the order Myxiniformes [42, 43] and the occurrence of 48 rods in the majority of fishes studied prior to 1967 led to the idea that the primitive karyotype of ancestral vertebrates evolved from chordate might consist of 48 rods [41]. Therefore, most subsequent researchers assumed karyotypic evolution in different groups of fishes to be founded on the basic assumption of 48 rods as the primitive number [41]. However, the discovery of 2n=24 rods in two species of freshwater eels [44, 45], 2n=36 rods in two species of *Myxine* and low diploid numbers ranging between 14- 42 in a large number of fish families showing an NF less than 36 in some cases [41] would possibly call for a more cautious prediction of the primitive karyotype of fish. The karyotype formulas of these loaches were found to be different, being 4m + 40sm + 6st in *C. linea*; 10m +26sm + 14st in *O. persa* and 18m +24sm + 8st in *O. tongiorgii.* The chromosome arm number (NF) of *C. linea (*94) was larger than that of the three steam loaches. Chromosome arm numbers of 66-152 have been reported for different species of the genus *Cobitis* [38]. In the present study, no cytological evidence was found for sex chromosome dimorphism in any of these four loaches, which agrees with reports on many other fish species [9-11]. In marine fishes too, despite the large number of living species, the occurrence of cytologically differentiated sex chromosomes appears to be rare [20].

The main obstacles in the study of nemacheilid loaches of the Middle East, including Iran, are the confused definitions of the genera and the large number of poorly diagnosed species described from this area [17, 46]. For a long time, the loaches of the Kor river basin have been considered to belong to two genera with four species:

*Cobitis linea*, *Orthrias persus* (Heckel, 1846), *Orthrias farsicus* (Nalbant & Bianco, 1998) and *Seminemacheilus tongiorgii* (Nalbant & Bianco, 1998). Stoumboudi et al. (2006) [47] and Prokofiev (2009) [48] placed most nemacheilid loaches from Eastern Europe and the Middle East in the genus *Oxynoemacheilus*. Recently, Freyhof et al. (2011) [17] reviewed the western Palaearctic *Oxynoemacheilus,* and Kottelat (2012) [46] listed species of this genus based mostly on the proposals of Prokofiev (2009) [48]. Freyhof et al. (2011) [17] transferred* Seminemacheilus *and* Orthrias *to the genus* Oxynoemacheilus *and considered* Orthrias persus* (Heckel, 1846), *Orthrias farsicus* (Nalbant & Bianco, 1998) and *Seminemacheilus tongiorgii *as synonymous to *Oxynoemacheilus persa *(Heckel, 1848) and *Oxynoemacheilus tongiorgii* (Nalbant & Bianco, 1998), respectively [1, 13, 18]. Maximum likelihood estimations of the phylogenetic relationships based on the COI barcode region clearly separate the three Iranian loach species from the Kor drainage and support their independent evolution from other studied loaches. This supports Freyhof et al. (2011) [17] and Kottelat’s (2012) [46] notion of the validity of *Oxynoemacheilus tongiorgii* and *Oxynoemacheilus persa*.

## Notes

**Acknowledgments: **The authors thank Shiraz University for financial support and J. Freyhof for his comments and the *C. linea* photo.
